# Clozapine-associated adverse drug reactions in 38,349 psychiatric inpatients: drug surveillance data from the AMSP project between 1993 and 2016

**DOI:** 10.1007/s00702-024-02818-7

**Published:** 2024-08-13

**Authors:** Lene Bleich, Renate Grohmann, Waldemar Greil, Dominik Dabbert, Andreas Erfurth, Sermin Toto, Johanna Seifert

**Affiliations:** 1 Department of Psychiatry, Social Psychiatry and Psychotherapy, Hannover Medical School, 30625 Hannover, Germany; 2 University Medical Center Göttingen, 37075 Göttingen, Germany; 3 Department of Psychiatry and Psychotherapy, LMU University Hospital, 80336 Munich, Germany; 4 Psychiatric Private Hospital, Sanatorium Kilchberg, 8802 Kilchberg, Switzerland; 5 Department of Forensic Psychiatry and Psychotherapy, Klinik Bremen-Ost, 28325 Bremen, Germany; 6 1st Department of Psychiatry and Psychotherapeutic Medicine, Klinik Hietzing, 1130 Vienna, Austria

**Keywords:** Adverse drug reaction, Clozapine, Drug safety, Pharmacovigilance, AMSP program, Psychiatric inpatients

## Abstract

Clozapine is a second-generation antipsychotic drug that offers superior treatment results in patients with schizophrenia but is also associated with significant risks. This study analyzes data on pharmacotherapy with clozapine and the associated adverse drug reactions (ADRs) in an inpatient setting including 38,349 patients. Data about the use of clozapine and reports of severe ADRs within the period 1993–2016 were obtained from the multicentered observational pharmacovigilance program “Arzneimittelsicherheit in der Psychiatrie” (AMSP). In total, 586 severe clozapine-associated ADRs were documented (1.53% of all patients exposed). Patients aged ≥65 years had a higher risk of ADRs than patients aged <65 years (1.96 vs. 1.48%; *p = *0.021). Significantly more ADRs were attributed to clozapine alone (396; 67.6% of all 586 ADRs) than to a combination with other drugs. The most frequent ADRs were grand mal seizures (0.183% of all 38,349 patients exposed), delirium (0.180%), increased liver enzymes (0.120%), and agranulocytosis (0.107%). We detected 24 cases (0.063%) of clozapine-induced extrapyramidal symptoms, of which 8 (0.021%) were attributed to clozapine alone. Five ADRs resulted in death (0.013%): 2 due to agranulocytosis (41 cases total) (mortality = 4.88%) and 3 due to paralytic (sub)ileus (16 cases) (mortality = 18.75%). The median dose of clozapine in all patients treated was 300 mg/day, in patients who developed ADRs 250 mg/day. The main risk factor for an ADR was pre-existing damage of the affected organ system. Overall, the results of this study highlight the importance of alertness—especially of frequently overlooked symptoms—and appropriate monitoring during treatment with clozapine, even at low doses.

**Supplementary Information:**

The online version contains supplementary material available at 10.1007/s00702-024-02818-7.

## Introduction

Schizophrenia is a severe psychiatric disorder characterized by positive and negative symptoms and cognitive impairments. It is one of the 25 most common causes of disability worldwide (Global Burden of Disease Study 2013 Collaborators 2015). This demonstrates the importance of an appropriate treatment of this disease. Antipsychotics are primarily used to treat schizophrenia, mania, and psychotic symptoms due to other illnesses. Antipsychotics are commonly categorized into “first-generation antipsychotics” (FGAs) and “second-generation antipsychotics” (SGAs). FGAs are effective in the treatment of the so called “positive symptoms” such as hallucinations and delusions due to their high antagonistic affinity for dopamine receptors of the D2 subtype (D2-receptors) (Farde et al. 1988; Kapur et al. 2006; Moschny et al. 2021). The possible occurrence of ADRs attributed to this D2-receptor blockade such as extrapyramidal symptoms (EPS) must be considered during prescription. The high risk of EPS and tardive dyskinesia associated with FGAs have contributed to their declining utilization over the years (Leslie and Rosenheck 2002; Toto et al. 2019). SGAs, on the other hand, have a lesser risk of causing EPS and tardive dyskinesia. This has been associated with their comparatively lower affinity for D2 but higher affinities for D1, D4 and D5 receptors as well as for numerous serotonergic (especially the 5-HT2A receptor), adrenergic, histaminergic, and muscarinic receptors (Meltzer et al. 1989; Jann et al. 1993; Moschny et al. 2021). However, the considerable risks of treatment with SGAs should not be neglected, as these drugs can also be associated with increased mortality and sometimes life-threatening adverse drug reactions (ADRs).

Clozapine was the first SGA and following its discovery, numerous other SGAs such as quetiapine, olanzapine, and risperidone followed (Leucht et al. 2013; Moschny et al. 2021). Over the last 20 to 25 years, SGAs have gradually replaced FGAs in everyday clinical practice (Leslie and Rosenheck 2002; Gill et al. 2005). Clozapine launched in the 1970s to the pharmacological market and shaped the term “atypical antipsychotic” or “SGA”. Despite the remarkable chemical, pharmacological and clinical heterogeneity, this term has since been generally applied to other antipsychotics (Möller 2000). Clozapine currently is the “gold standard” in treatment of treatment-resistant schizophrenia and is approved almost exclusively for this area of application (Conley and Kelly 2001; Moore et al. 2007; McIlwain et al. 2011), an example for an exception is the possible treatment of dyskinesia in Parkinson’s disease with clozapine (Fox et al. 2018) and beneficial efficacy outcomes in Parkinson’s disease psychosis (Wagner et al. 2021). Clozapine has a high affinity for D1, D4, serotonin, and α receptors and a comparatively low affinity for D2-receptors. Its high antipsychotic efficacy is primarily due to its affinity for the D4 receptor, whereas the much rarer occurrence of EPS (Schmauss et al. 1989) is attributed to the only mild D2-receptor blockade (Farde et al. 1988; Meltzer et al. 1989; Kapur et al. 2006). Further advantages of clozapine-treatment are a reduction in negative symptoms, comorbid substance use, aggression, and suicidality (Meltzer et al. 2003; Frogley et al. 2012; van der Zalm et al. 2021), as well as improved treatment adherence and patient satisfaction (McEvoy et al. 2006; Stroup et al. 2016).

Since the frequent occurrence of deaths associated with agranulocytosis shortly after the market launch in 1972 (Amsler et al. 1977), the prescription of clozapine has been subject to special conditions. However, agranulocytosis is by far not the only concerning ADR under clozapine with potentially life-threatening sequelae. Although rare, ADRs such as epileptic seizures, delirium, elevated liver enzymes, myocarditis and paralytic ileus are also relevant (Nielsen et al. 2013). Due to the severe ADRs and the more complicated handling of clozapine with obligatory blood cell monitoring, the use of clozapine in clinical practice is often conservative and cautious and often used much later than indicated despite its outstanding efficacy (Wheeler 2008). Early and consistent ADR management and pharmacovigilance are mandatory in the clinical treatment. Therefore, this study analyses the clozapine associated ADRs in an inpatient setting, which are highly relevant for its everyday clinical use. The inpatient data consists of a total of 38,349 patients treated with clozapine during the study period, with 586 documented ADRs attributed to clozapine. This study, therefore, encompasses a much larger and, to our knowledge, unprecedented sample compared to previously published studies. This should provide a more realistic and naturalistic picture of severe clozapine-associated ADRs, of which some are often overlooked in everyday clinical practice. Our aim is to bridge this knowledge gap, clarifying the use of clozapine and the necessity for monitoring of specific ADRs to enhance patients’ safety.

## Methods

### The AMSP program

Data on the prescription of clozapine and reports of severe ADRs within the period 1993–2016 were obtained from the database of the Drug Safety Program in Psychiatry (German: “Arzneimittelsicherheit in der Psychiatrie”; AMSP) which is a multicentered observational pharmacovigilance program that has been collecting data about psychotropic drug use in psychiatric inpatients since 1993. More than 100 hospitals from Germany, Austria, and Switzerland have participated in the ongoing AMSP-project (Grohmann et al. 2014). In our study-period, the AMSP program gathered reports from 107 hospitals, 67 of which reported clozapine associated ADRs.

The AMSP program records an ADR if it’s “severe”, newly occurring, unusual, occurring during discontinuation of the drug, or in the context of pharmacokinetic interactions. According to AMSP guidelines, an ADR is considered severe if one of the following reasons is met: (1) immediate threat of the event, (2) relevant impairment of the patient’s everyday functions, (3) creating the need for special measures (transfer, surgery), or (4) (for some ADRs) exceeding a quantitative limit (e.g. a drop in neutrophil granulocytes to below 1.500/µl). For each organ system the AMSP protocol provides additional guidelines to further coordinate ADRs and standardize classification (Grohmann et al. 2014).

### Assessment and recording of ADRs

To record ADRs, physicians in participating hospitals act as “drug monitors” who document ADRs on standardized questionnaires where the symptoms and classification as “severe” are described in detail. The questionnaires also include age, sex, psychiatric and somatic diagnosis, dosage of the medication, a probability grading (see below), alternative hypotheses, patient-related risk factors, measures taken to treat the ADR, course, and possible previous exposures to the drug as well as supplementary data on laboratory, technical examinations, etc. After the patient’s written consent, the documented cases are re-examined for plausibility by a senior physician of the hospital. In addition to the ADRs, the number of inpatients and patients treated with the respective medication is also documented in the AMSP program. The diagnoses are documented in AMSP as assessed clinically by the treating physicians according to the International Classification of Disease, 10th Version (ICD-10).

The cases are then discussed in regional case conferences, where the documentation forms are completed with further relevant information and sent to the data collection center. There, every case is checked for completeness and plausibility by an experienced psychiatrist of the AMSP program. Twice a year, a selection of cases (e.g., of high clinical interest, severe or unusual cases) is discussed in detail at central case conferences. The cases are critically analyzed in a demographic and data-based context by experts.

### Probability grading and categorization of ADRs

The AMSP-program assesses the probability with which an ADR can be attributed to a specific drug. Therefore, the ADRs are differentiated according to the so-called “probability grades” (“W grades”) defined by the AMSP program (Grohmann et al. 2004, 2014):

- W grade 1 = possible: The ADR is unusual for the drug or its dose, has an unusual time course or the probability of another cause for the ADR is greater than 50%.
- W grade 2 = probable: The probability of another cause is less than 50%, the ADR matches the time course and is known for the drug.
- W grade 3 = definite: fulfillment of the W2 criteria plus re-occurrence of the ADR on re-exposure with the drug.
- W grade 4 = questionable or not sufficiently documented: Causal relationship between the ADR and the drug are not assessable or unclear. The ADR needs to be recorded due to clinical importance, e.g. deaths.

The ADRs analyzed in this study are severe ADRs which have been rated as grade 2 or 3 in the context of clozapine probability.

In this study, the ADRs in clozapine users were further subdivided according to the affected organ system and clinical presentation.

Often, patients are treated with more than one drug. In these cases, multiple drugs can be imputed and be held as responsible for having caused an ADR. Each individual drug then goes through the probability grading process described above. In this study therefore, the ADRs are divided into three groups of imputations: If clozapine alone is held to be responsible for the occurrence of a certain ADR, this is referred to as “imputed alone”. If another drug or several other drugs are causally linked to the ADR in addition to clozapine, the term “imputed in combination” is used. In addition to these two, the term “all cases” refers to both, imputed alone and in combination.

### Statistical analysis

The main part of the statistical analysis was carried out with Excel^©^. ADRs attributed to clozapine were reported as absolute numbers and percentages of the total quantity of ADRs of the respective form of imputation as well as of the total number of patients treated with clozapine. The most frequent ADRs (ADRs which accounted for more than 10% of all 586 documented ADRs) were subject to further statistical tests in the means of chi-square tests and calculations of odds ratios (ORs) and 95% confidence intervals (CIs). The tests were restricted to comparison of the ADR frequency related to age, and sex. The significance level was set to 5% (*p > *0.05).

## Results

### Data on patients treated with clozapine

Between 1993 and 2016, a total of 495,615 psychiatric inpatients were observed as part of the AMSP project. 333,175 of these patients (67.2%) were treated with antipsychotics and 38,349 (7.7%) with clozapine. In 33,573 (87.6% of 38,349) of the patients treated with clozapine, schizophrenia was the patient’s primary diagnosis. Other primary diagnoses as a reason for the current hospitalization such as depression (4.9%), organic disorders (2.9%), neuroses or personality disorders (1.8%), mania (1.6%), and addiction (0.4%) were less frequent.

A majority of patients treated with clozapine were younger than 65 years (34,471 patients; 89.9%) and 3878 patients (10.1%) were 65 years old or older. Therefore, significantly more younger than older patients were treated with clozapine (*p < *0.001). 20,939 (54.6%) of the clozapine-treated patients were male, 17,410 (45.4%) were female. Thus, significantly more men than women were treated with clozapine (*p < *0.001).

### Demographic data of patients with clozapine-associated ADRs

In total 586 ADRs (1.53% of all patients treated with clozapine) were documented. 396 cases (67.6%) were attributed to clozapine alone (“single imputations”) and 190 (32.4%) were attributed to a combination of drugs including clozapine ("multiple imputations”).

Table 1 shows the age and sex of patients who developed an ADR (all cases and single imputations. A significant sex difference in the occurrence of both all clozapine-associated ADRs and those with multiple imputations could not be observed. In 510 of the 586 ADRs cases, the patients were between 18 and 64 years old (87.0%). Overall, patients aged ≥65 years had a significantly higher risk of clozapine-associated ADRs in general than younger patients (*p = *0.021) and when clozapine was imputed in combination (*p = *0.018). However, there was no significant difference between the two age groups when clozapine was imputed alone (*p > *0.05).

**Table 1:** Age and sex of patients treated with clozapine and patients who developed an ADR

	Patients treated with CLZ; *N* = 38,349	ADRs, “all cases” (% of *N*)	*p*-value	ADRs, CLZ imputed alone (% of *N*)	*p*-value	ADRs, CLZ imputed in combination (% of *N*)	*p*-value
Sex							
Male	20,939	329 (1.57%)	0.450	219 (1.05%)	0.778	110 (0.53%)	0.361
Female	17,410	257 (1.48%)		177 (1.02%)		80 (0.46%)	
Age							
<65	34,471	510 (1.48%)	0.021	349 (1.01%)	0.244	161 (0.47%)	0.018
≥65	3878	76 (1.96%)		47 (1.21%)		29 (0.75%)	

^*CLZ*clozapine, *N*number (of cases), *ADR*adverse drug reaction^

### Clozapine-associated ADRs

#### Type and clinical presentation of clozapine-associated ADRs

**Fig. 1:**
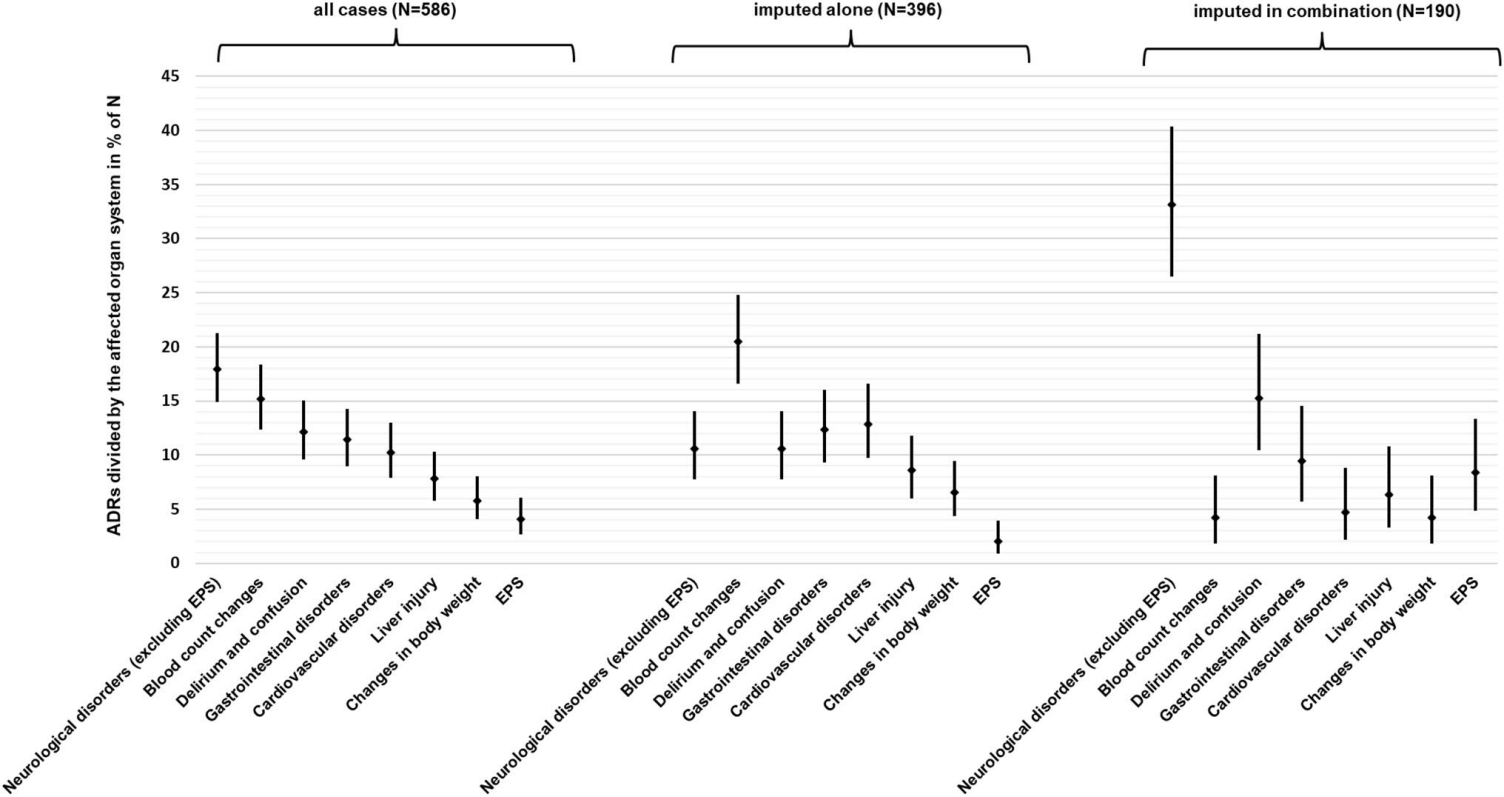
Most common ADRs (all cases, imputed alone, and imputed in combination) according to the affected organ system. *N* number (of cases), *ADR* adverse drug reaction, *EPS* extrapyramidal symptoms

Table 2 lists all the 586 ADRs that were causally linked to clozapine subdivided by the affected organ system. Regarding all cases, neurological disorders excluding EPS occurred most frequently (17.9% of all ADRs) followed by blood count changes (15.2%). Other common ADRs were delirium and confusion (12.1%), gastrointestinal disorders (11.4%), and cardiovascular disorders (10.2%). A total of 396 ADRs imputed only clozapine, i.e., 67.6% of all clozapine-associated ADRs. The most common ADRs in which clozapine was imputed alone were blood count changes (20.5% of the 396 ADRs imputed alone), cardiovascular disorders (12.9%), and gastrointestinal disorders (12.4%). Figure 1 shows a comparison of the most common ADRs (all cases, single imputations, and multiple imputations).

**Table 2:** Occurrence of clozapine-associated ADRs (all cases and single imputations) according to the affected organ system

Organ system affected/type of ADR	All cases of CLZ-associated ADRs			ADRs with single imputation of CLZ		
	All cases (*N* = 586)	Frequency in relation to all cases in % (*N* = 586)	Frequency in relation to all patients treated with CLZ (*N* = 38,349) in %	CLZ imputed alone (*N* = 396)	Frequency in relation to cases “imputed alone” in % (*N* = 396)	Frequency in relation to all patients treated with CLZ (*N* = 38,349) in %
Neurological disorders (excluding EPS)	105	17.9	0.274	42	10.6	0.110
Blood count changes	89	15.2	0.232	81	20.5	0.211
Delirium and confusion	71	12.1	0.185	42	10.6	0.110
Gastrointestinal disorders	67	11.4	0.175	49	12.4	0.128
Cardiovascular disorders	60	10.2	0.156	51	12.9	0.133
Liver injury	46	7.8	0.120	34	8.6	0.089
Changes in body weight	43	7.3	0.112	30	7.6	0.078
EPS	24	4.1	0.063	8	2.0	0.021
Urological disorders	20	3.4	0.052	13	3.3	0.034
Mental disorders, non-delirious	19	3.2	0.050	9	2.3	0.023
Metabolic and electrolyte disorders	7	1.2	0.018	5	1.3	0.013
Skin conditions	12	2.0	0.031	12	3.0	0.031
Temperature dysregulation	9	1.5	0.023	9	2.3	0.023
Interaction causing intoxication	5	0.9	0.013	4	1.0	0.010
Allergic reaction (not skin)	4	0.7	0.010	4	1.0	0.010
Respiratory disorders	2	0.3	0.005	1	0.3	0.003
Other non-specific symptoms	1	0.2	0.003	1	0.3	0.005
Muscle disorders	1	0.2	0.003	0	0.0	0.000
Disorders of genital functions	1	0.2	0.003	1	0.3	0.003

*CLZ* clozapine, *N* number of cases, *ADR* adverse drug reaction, *EPS* extrapyramidal symptoms

**Fig. 2:**
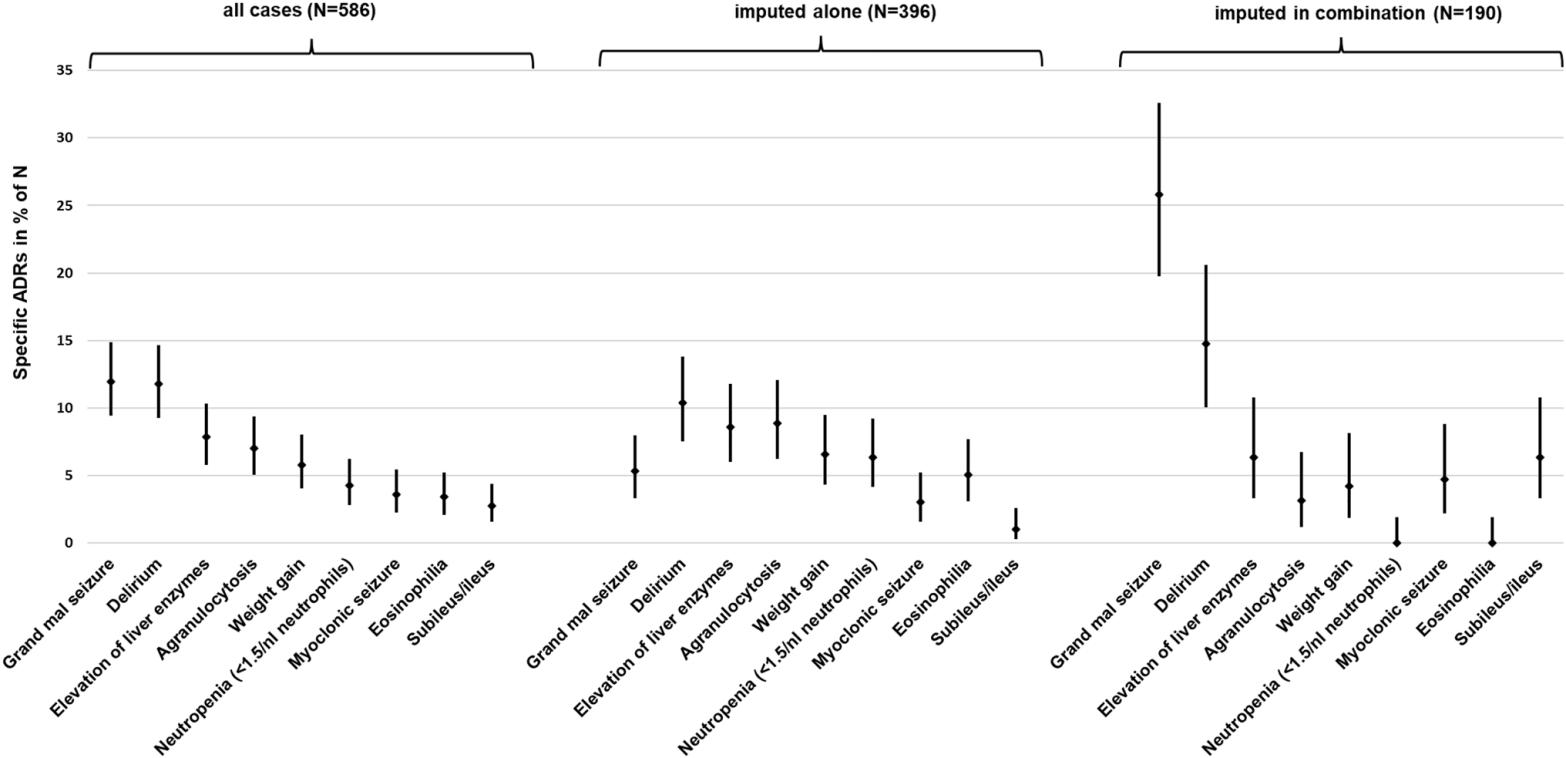
Most common specific clozapine-associated ADRs (all cases, imputed alone, and imputed in combination). *N* number (of cases), *ADR* adverse drug reaction

The ADRs were further specified according to the specific clinical presentation of the ADR (Table 3; Fig. 2). By far the most common ADRs associated with clozapine were grand mal seizures (12.0% of all ADRs) and delirium (11.8%). Liver enzyme elevations (7.9%) and agranulocytosis (7.0%) were also common. The most common ADRs with clozapine in single imputation were delirium (10.35% of “imputed alone” cases), agranulocytosis (8.8%), and elevated liver enzymes (8.6%).

**Table 3:** Occurrence of specific clozapine-associated ADRs (all cases and single imputations)

Clinical presentation of the ADR	All cases of CLZ-associated ADRs			ADRs with single imputation of CLZ		
	All cases; *N* = 586	Frequency in relation to all cases in % (*N* = 586)	Frequency in relation to all patients treated with CLZ (*N* = 38,349) in %	CLZ imputed alone; *N* = 396	Frequency in relation to cases “imputed alone” in % (*N* = 396)	Frequency in relation to all patients treated with CLZ (*N* = 38,349) in %
Neurological ADRs (excluding EPS)						
Grand mal seizure	70	11.9	0.183	21	5.3	0.055
Myoclonic seizure	21	3.6	0.055	12	3.0	0.031
Epileptic seizure, not grand mal	8	1.4	0.021	4	1.0	0.010
Speech disorders/stuttering	3	0.5	0.008	3	0.8	0.008
Ataxia	2	0.3	0.005	1	0.3	0.003
Dizziness	1	0.2	0.003	1	0.3	0.003
Blood count changes						
Agranulocytosis (≤0.5/nl neutrophils)	41	7.0	0.107	35	8.8	0.091
Neutropenia (<1.5/nl and >0.5/nl neutrophils)	25	4.3	0.065	25	6.3	0.065
Eosinophilia (>1.5/nl eosinophils)	20	3.4	0.052	20	5.1	0.052
Thrombocytopenia	2	0.3	0.005	1	0.3	0.003
Neutro- and thrombocytopenia	1	0.2	0.003	0	0.0	0.000
Mental disorders (Delirium and confusion)						
Delirium	69	11.8	0.180	41	10.4	0.107
Confusion	2	0.3	0.005	1	0.3	0.003
Gastrointestinal disorders						
Hypersalivation	19	3.2	0.050	15	3.8	0.039
Pancreatitis	17	2.9	0.044	17	4.3	0.044
(Sub-)ileus	16	2.7	0.042	4	1.0	0.010
Severe constipation	4	0.7	0.010	2	0.5	0.005
Parotitis	3	0.5	0.008	3	0.8	0.008
Lipase/amylase increase (>3 times the norm)	3	0.5	0.008	3	0.8	0.008
Nausea (>1 week)	2	0.3	0.005	2	0.5	0.005
Acute cholecystitis	1	0.2	0.003	1	0.3	0.003
Gastrointestinal complaints	1	0.2	0.003	1	0.3	0.003
Lymphocytic colitis	1	0.2	0.003	1	0.3	0.003
Cardiovascular disorders						
Myocarditis/pericarditis	20	3.4	0.052	20	5.1	0.052
Sinus tachycardia (heart rate >120/min)	14	2.4	0.037	12	3.0	0.031
Syncope	13	2.2	0.034	10	2.5	0.026
Hypotension/dizziness (<90 mmHg)	4	0.7	0.010	2	0.5	0.005
QT-prolongation (>500 ms)	3	0.5	0.008	2	0.5	0.005
Arrhythmias	3	0.5	0.008	2	0.5	0.005
Cardiomyopathy	2	0.3	0.005	2	0.5	0.005
Thrombosis	1	0.2	0.003	1	0.3	0.003
Liver injury						
Elevated liver enzymes (>5 times the norm)	46	7.8	0.120	34	8.6	0.089
Changes in bodyweight						
Weight gain (≥+10%)	34	5.8	0.089	26	6.6	0.068
Weight gain with metabolic syndrome	4	0.7	0.010	3	0.8	0.008
Weight gain with hyperlipidemia	3	0.5	0.008	0	0.0	0.000
Weight gain, followed by diabetes	2	0.3	0.005	1	0.3	0.003
EPS						
Pisa/metronome syndrome	21	3.6	0.055	7	1.8	0.018
Akathisia	2	0.3	0.005	1	0.3	0.003
Early dyskinesia	1	0.2	0.003	0	0.0	0.000
Urological disorders						
Enuresis nocturna	11	1.9	0.029	7	1.8	0.018
Incontinence	7	1.2	0.018	6	1.5	0.016
Urinary retention	2	0.3	0.005	0	0.0	0.000
Mental disorders, non-delirious						
Quantitative disturbance of consciousness	8	1.4	0.021	3	0.8	0.008
Sedation	8	1.4	0.021	3	0.8	0.008
Aggression	2	0.3	0.005	2	0.5	0.005
Compulsive symptoms	1	0.2	0.003	1	0.3	0.003
Metabolic and electrolyte disorders						
First manifestation of diabetes mellitus	4	0.7	0.010	4	1.0	0.010
Treatment-related hyperlipidemia	1	0.2	0.003	1	0.3	0.003
Polydipsia	1	0.2	0.003	0	0.0	0.000
Galactorrhea	1	0.2	0.003	0	0.0	0.000
Skin conditions						
Allergic skin reaction/exanthema/vasculitis	5	0.9	0.013	5	1.3	0.013
Allergic reaction skin + other organs	4	0.7	0.010	4	1.0	0.010
Edema	3	0.5	0.008	3	0.8	0.008
Temperature dysregulation						
Fever (>39 °C)	9	1.5	0.023	9	2.3	0.023
Interaction causing intoxication						
Drug interaction causing intoxication	5	0.9	0.013	4	1.0	0.010
Allergic reaction (not skin)						
Allergic reaction (not skin)	4	0.7	0.010	4	1.0	0.010
Respiratory disorders						
Shortness of breath (subjective)	1	0.2	0.003	1	0.3	0.003
Pneumonia	1	0.2	0.003	0	0.0	0.000
Muscle/movement disorders						
Increase in creatinine kinase	1	0.2	0.003	0	0.0	0.000
Disorders of genital functions						
Priapism	1	0.2	0.003	1	0.3	0.003
Other non-specific symptoms						
Fall of unknown origin	1	0.2	0.003	1	0.3	0.003

*CLZ*  clozapine, *N *number of cases, *ADR* adverse drug reaction, *EPS* extrapyramidal symptoms

#### Drugs imputed in clozapine-associated ADRs with multiple imputations

The most frequently used specific drug combinations imputed in the 190 ADRs in multiple imputations are briefly mentioned here. 26 ADRs were documented (13.7% of the 190 ADRs) in which both clozapine and haloperidol were imputed. 25 ADR cases (13.2%) occurred in combination with lithium, 19 (10.0%) in combination with valproic acid. In 16 cases (8.4%), the combination of clozapine and drugs for the treatment of gastrointestinal complaints was imputed. More than 10 ADR cases occurred under combination with each amisulpride (6.8%), chlorprothixene (6.3%), risperidone (5.8%), and levomepromazine (5.8%). In 6 ADR cases, 5 of which presented as epileptic seizures and as ileus in one case, a triple combination of clozapine, haloperidol, and biperiden was imputed. Overall, most ADRs with multiple imputations occurred under combination of clozapine with other SGAs (46 cases, 24.2%) or with butyrophenones (48 cases, 25.3%).

#### Comparison of the ADRs regarding the different imputations

The most common ADRs were subject to further analyses, particularly in comparison of the different imputations. The most important results are briefly described below and presented in Fig. 1 and Fig. 2. In general (considering all cases), ADRs were significantly more often attributed to clozapine alone than to a combination with other drugs (*p < *0.001).

When looking at the ADR frequencies subdivided by the affected organ system (Fig. 1), neurological disorders without EMPS occurred significantly more frequently when clozapine was imputed in combination (33.1% of “imputed in combination” cases) than when imputed alone (10.6% of “imputed alone” cases; *p < *0.001). EPS also occurred significantly more frequently under combination imputation (8.4%) than sole imputation of clozapine (2.0%; *p < *0.001). In contrast, there were significantly more cases of blood count changes with clozapine being imputed alone (20.5%) than imputed in combination with other drugs (4.2%; *p < *0.001). Cardiovascular disturbances also were significantly more frequently observed when clozapine was imputed alone (12.9%) than in combination (4.7%; *p = *0.002).

Regarding the specific ADRs (Fig. 2) grand mal seizures appeared significantly more often with clozapine being imputed in combination (25.8%) than alone (5.3%; *p < *0.001). (Sub-)ileus also occurred significantly more often when clozapine was imputed in combination (6.3%) than alone (1.0%; *p < *0.001). In contrast, agranulocytosis occurred significantly more frequently with clozapine alone being imputed (8.8%) than in combination (3.2%; *p = *0.012). Neutropenia (6.3%) occurred exclusively when clozapine was imputed alone, and therefore significantly more often than under combination imputation (*p < *0.001). Same applies to eosinophilia (5.1%; *p = *0.002).

#### Analyses of the most common ADRs in relation to sex and age

Table 4 lists the most common ADRs according to sex and age of the affected patients.

**Table 4:** Most common clozapine-associated ADRs (all cases) in relation to sex and age

	*N* ADRs	Sex				Age			
		Men (% of all men with an ADR)	Women (% of all women with an ADR)	*p*-value	OR, 95% CI	< 65 years (% of all ADR-patients <65)	≥ 65 years (% of all ADR-patients ≥ 65)	*p*-value	OR, 95% CI
*N* ADRs	586	329 (100%)	257 (100%)			510 (100%)	76 (100%)		
Neurological disorders (excluding EPS)	105	60 (18.24%)	45 (17.51%)	0.820	OR = 1.05; CI: 0.69–1.61	98 (19.22%)	7 (9.21%)	0.034	OR = 2.34; CI: 1.05–5.26
Blood count changes	89	40 (12.16%)	49 (19.07%)	0.021	OR = 0.59; CI: 0.37–0.93	75 (14.71%)	14 (18.42%)	0.400	OR = 0.76; CI: 0.41–1.43
Delirium and confusion	71	45 (13.68%)	26 (10.12%)	0.190	OR = 1.41; CI: 0.84–2.35	55 (10.78%)	16 (21.05%)	0.011	OR = 0.45; CI: 0.24–0.84
Gastrointestinal disorders	67	43 (13.07%)	24 (9.34%)	0.159	OR = 1.46; CI: 0.86–2.48	59 (11.57%)	8 (10.53%)	0.790	OR = 1.11; CI: 0.51–2.43
Cardiovascular disorders	60	43 (13.07%)	17 (6.61%)	0.011	OR = 2.12; CI: 1.18–3.82	53 (10.39%)	7 (9.21%)	0.751	OR = 1.14; CI: 0.50–2.62
Grand mal seizures	70	42 (12.77%)	28 (10.89%)	0.488	OR = 1.20; CI: 0.72–1.99	65 (12.75%)	5 (6.58%)	0.112	OR = 2.07; CI: 0.81–5.33

*N* number (of cases), *ADR* adverse drug reaction, *EPS* extrapyramidal symptoms, *OR* odds ratio, *CI* confidence interval

**Sex**—Women were significantly more frequently affected by blood count changes than men (*p = *0.021; OR = 0.59; CI: 0.37–0.93). In contrast, significantly more cardiovascular disorders occurred in men than in women (*p = *0.011; OR = 2.12; CI: 1.18–3.82).

**Age**—Differences related to age were only found for neurological disorders and for delirium and confusion. Regarding differences related to age, neurological disorders affected significantly more often younger patients (<65 years) than older patients (≥65 years; *p = *0.034; OR = 2.34; CI: 1.05–5.26). Delirium and confusion affected older patients significantly more often than younger patients (*p = *0.011; OR = 0.45; CI: 0.24–0.84).

### Dosage of clozapine

The median dose in all patients treated with clozapine was 300 mg/day. The median dose in patients who developed a clozapine-related ADR (imputed alone) was 250 mg/day. The median dose in all the most common types of clozapine-associated ADRs (listed in Table 5) was less than 450 mg/day. The highest median doses were found in Pisa syndromes (412.5 mg/day) and grand mal seizures (400 mg/day). The exact median doses for the most frequent ADRs associated with clozapine are listed in Table 5.

**Table 5:** Dosage of clozapine (mg/day) amongst the most common clozapine-associated ADRs (all cases)

ADR	Cases (all cases)	Median dose of clozapine in mg/day
Grand mal seizure	70	400
Delirium	69	200
Elevation of liver enzymes	46	200
Agranulocytosis/neutropenia	66	300
Weight gain	43	250
Myoclonic seizure	21	300
Eosinophilia	20	175
Myocarditis/pericarditis	20	225
Hypersalivation	19	275
Pisa syndrome	18	412.5
Pancreatitis	17	225
Subileus/ileus	16	262

*ADR* adverse drug reaction

### Risk factors for the development of ADRs under clozapine treatment

In slightly more than half of the ADR-patients (326, 55.6% of all ADR cases) no risk factors were documented. At least one risk factor was documented in 260 patients (44.4%), with the most common risk factor being previous damage to the affected organ system in 114 patients (19.5%), most often in patients with a toxic delirium (*N* = 22), grand mal seizures (*N* = 18), or a Pisa syndrome (*N* = 9) including cerebral atrophy, cerebrovascular lesions, dementia, hypoxic perinatal, or traumatic cerebral damage as the most common pre-existing impairments. Other risk factors, all of which accounted for less than 10% of the ADRs, were ADR sensitivity, rapid dose titration, reduction of dose or discontinuation, infection, change in smoking habits, high starting dose, previous treatment with other antipsychotics, and harmful use/addiction. Risk factors were by far most often documented in ADR cases of delirium: Risk factors were present in 50 of 69 cases of delirium (72.5% of all delirium cases).

### Measures taken to counteract the ADRs and course of the ADRs

**Countermeasures**—Clozapine was discontinued in 393 cases (67.1% of all ADR cases). The dose of clozapine was reduced in 153 patients (26.1%). In only 13 cases (2.2%), clozapine treatment was continued at the same dosage. In 136 patients with ADRs (23.2%), other specialists were consulted, 107 patients (18.3%) were transferred to another specialty department. Drugs to counteract the ADR were used in 214 of the ADR cases (36.5%), in 119 patients (20.3%) the ADR was counteracted by non-pharmacological measures. In 17 ADR cases (2.9%) no specific measures were taken or documented.

**Course of the ADR**—In 447 cases (76.3%), the clozapine-associated ADR subsided completely. In 92 patients (15.7%), the ADR was reported to be in remission at the end of the observation period, whereas in 40 cases, the ADR remained stable/unchanged. In 2 cases (0.3%) the further course was unknown. In 5 cases, the affected patient (0.9%; 5 out of 586) died because of the ADR.

### Clozapine-associated ADRs resulting in death

**Agranulocytosis**—Among the 5 deaths (0.013% of all 38,349 patients treated with clozapine), 2 were the result of agranulocytosis. With a total of 41 cases of clozapine-induced agranulocytosis, the mortality of this ADR was 4.9%. In both cases, clozapine was imputed in combination with other psychotropic drugs, in one case with mirtazapine, in the other with quetiapine.

**(Sub-)Ileus**—In 3 patients, a paralytic (sub-)ileus resulted in the affected patient’s death. With a total of 16 cases of clozapine-induced (sub-)ileus, this implies a mortality of 18.8%. In all cases of fatal (sub-)ileus, the patients had pre-existing damage to the intestine and concomitantly used drugs with additive anticholinergic and/or constipating effects (i.e., pirenzepine, mebeverine, amitriptyline, perazine, biperiden, levomepromazine, chlorprothixene, haloperidol).

### Comparison of clozapine-associated ADR frequencies to ADR frequencies of other antipsychotics

**Fig. 3:**
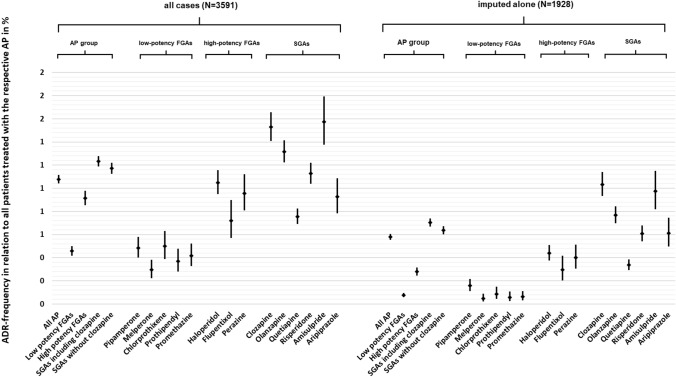
Comparison of ADR frequencies in relation to all patients treated with the respective antipsychotic (all cases, imputed alone). *N* number (of cases), *ADR* adverse drug reaction, *AP *antipsychotic, *FGA(s)* first-generation antipsychotic(s), *SGA(s)* second-generation antipsychotic(s)

Figure 3 shows the ADR rate of different antipsychotics in relation to the number of patients treated with the respective drug. A table with the exact data can be found in the supplementary material of this study.

A total of 333,175 patients were treated with antipsychotics in general of which 3591 (1.08%) developed an ADR. A majority of the reported ADRs were associated with the use of SGAs with 1.23% of all patients treated with SGAs developing an ADR.

Amisulpride was the antipsychotic with the highest ADR rate (1.57% of patients treated with amisulpride) closely followed by clozapine (1.53% of patients treated with clozapine). Overall, clozapine was therefore significantly more often imputed in ADRs than e.g., haloperidol (*p < *0.001), olanzapine (*p = *0.007), and risperidone (*p < *0.001). There was no significant difference between the ADR frequency of clozapine and amisulpride (*p = *0.705).

When considering “single imputation” ADRs, clozapine was most commonly imputed (1.03%) followed by amisulpride (0.97%) and olanzapine (0.77%). Clozapine-associated ADRs were therefore significantly more often observed than olanzapine-associated ones (*p < *0.001). In comparison to amisulpride, no significant difference was found (*p = *0.552).

## Discussion

Of the 38,349 patients receiving clozapine, 586 developed an ADR. Neurological disorders (excluding EPS) occurred most frequently followed by blood count changes. Other common ADRs were delirium and confusion, gastrointestinal disorders, and cardiovascular disorders. A total of 396 ADRs imputed only clozapine, 190 ADRs imputed multiple drugs including clozapine. In general (considering all cases), ADRs were significantly more often attributed to clozapine alone than to a combination with other drugs. Women were significantly more frequently affected by blood count changes than men. In contrast, significantly more cardiovascular disorders occurred in men than in women. Regarding differences related to age, neurological disorders affected younger patients (<65 years) than older patients (≥65 years) significantly more often. Delirium and confusion, however, affected older patients significantly more often than younger patients. Five deaths due to clozapine treatment were documented, 2 were the result of agranulocytosis and 3 of a paralytic (sub-)ileus. Therefore, we found the (sub-)ileus to be the most lethal ADR of clozapine.

When comparing the ADR rate of individual antipsychotics with one another, clozapine was the antipsychotic with the second highest occurrence of ADRs, only exceeded by amisulpride (although in a statistically insignificant manner). This is most likely due to the large number of reports of hyperprolactinemia and galactorrhea under treatment with amisulpride (Glocker et al. 2021), which is a fairly easily detectable and also less dangerous ADR than common severe ADRs due to clozapine such as a toxic delirium, grand mal seizures, or agranulocytosis/neutropenia.

### Clozapine-induced ADRs according to age and sex

Older patients may be more susceptible to ADRs, as has also been described for clozapine-associated ADRs (Ismail et al. 2012; Bishara and Taylor 2014). A frequently stated reason for this phenomenon is the increasing tendency towards polypharmacy in older patients (Spina and de Leon 2007). We found older patients to be significantly more often affected by an ADR when a combination of drugs was imputed and in contrast no significant age-difference when clozapine was imputed alone. Van der Horst et al. (2023), on the other hand, concluded that neither monotherapy nor polytherapy with clozapine increased the risk of ADRs. The same study found women to be significantly more often affected by ADRs than men (van der Horst et al. 2023), a finding which could not be replicated in our study, perhaps due to the different study designs. The study by van der Horst et al. (2023) had a cross-sectional design which limited the authors ability to establish causality between drugs and ADRs a limitation, which is not shared by the present study in which causality is meticulously assessed by the AMSP team members.

### Association of clozapine dosage and the occurrence of ADRs

In our study, patients with clozapine-associated ADRs were treated with a lower median dose of clozapine than what was used for all 38,349 patients (250 vs. 300 mg/day). Our results suggest that many clozapine-associated ADRs tend to occur at lower doses, such as during the titration phase, which is often performed during inpatient stay. The dose-dependency of clozapine-induced ADRs is not fully clarified yet (Chandru and Gunja 2023) and further studies are needed, as the data appears to be very limited (Siafis et al. 2023). However, in general, the individual plasma concentration of clozapine seems to be more relevant than the actual dose (Wohkittel et al. 2016; Siskind et al. 2021). A systematic review from 2022 found an association between ADRs and clozapine plasma levels for EEG abnormalities, heart rate variability, obsessive–compulsive symptoms, hyperinsulinemia, metabolic syndrome, and constipation. Therefore, they stated that therapeutic drug monitoring (TDM) of plasma clozapine concentrations has a significant benefit in clinical practice, as it improves drug safety and patient satisfaction (Skokou et al. 2022).

### Clozapine-associated ADRs

#### Epileptic seizures

In general, clozapine has a higher risk of seizures than other antipsychotics (Druschky et al. 2019). In the present study, grand mal seizures were found to be the most frequent clozapine-associated ADR. The risk for clozapine-induced seizures was significantly higher when other drugs that potentially lower the seizure threshold were concomitantly administered. A recent Japanese study on clozapine-induced epileptic seizures associated clozapine with an increased risk of seizures, especially at high doses (>400 mg/day) (Hatano et al. 2023). Of 1874 clozapine-associated ADRs, 11.04% were seizures (Hatano et al. 2023), very similar to our results. High doses, younger age, antipsychotic polypharmacy, and combination with lithium were also significantly associated with the occurrence of clozapine-induced seizures (Hatano et al. 2023). However, unlike Hatano et al. 2023, we were unable to confirm an age-dependent effect of clozapine-associated seizures. On the other hand, other studies found clozapine-induced electroencephalography (EEG) abnormalities to be more common in younger than in older patients (Kikuchi et al. 2014). It has been suggested that clozapine-induced seizures are not necessarily precipitated by nonspecific EEG abnormalities (Treves and Neufeld 1996; Chung et al. 2002). In general, minor EEG abnormalities in patients treated with clozapine are very common (Günther et al. 1993) and appear to correlate with the plasma clozapine concentration (Varma et al. 2011). Because these findings only rarely reveal evidence of seizure activity, routine EEG monitoring is not generally recommended (Kar et al. 2016). Moreover, seizures in patients treated with clozapine with or without a known seizure disorder can generally be efficiently managed with antiepileptics, therefore, they should not be considered a reason for clozapine discontinuation (Nielsen et al. 2013).

#### Cardiovascular disorders

In general, the use of clozapine cannot be causally linked to an increased mortality or morbidity due to cardiovascular complications in patients treated with clozapine (Joy et al. 2017). However, clozapine is one of the antipsychotics with the highest risk of cardiovascular ADRs (Friedrich et al. 2020) and potentially fatal cardiovascular clozapine-associated ADRs, such as clozapine-induced myocarditis and cardiomyopathy, though both rare, are well known. A meta-analysis from 2016 analyzed studies on cardiac ADRs due to clozapine and observed an incidence of myocarditis of <0.1–1% and of cardiomyopathy of <0.01–0.1% with an average mortality rate of 25% (Curto et al. 2016). Our study found the cardiovascular system to be the fifth most common affected organ system by clozapine-induced ADRs, however, no fatal outcomes were observed. Whether long-term consequences of the ADRs and associated mortality occurred after discharge could not be accurately assessed due to the exclusive clinical setting of our study. While we found that significantly more men than women were affected by clozapine-associated cardiovascular complications, data in the current literature on sex-differences in relation to these ADRs is lacking.

#### Neutropenia and agranulocytosis

The perhaps most prominent and most feared clozapine-induced ADR is agranulocytosis. Indeed, the risk of agranulocytosis of clozapine by far exceeds that of other antipsychotics and is highest within the first 3 months of treatment (Glocker et al. 2023). In 2018, a meta-analysis including 108 studies on the epidemiology of clozapine-associated neutropenia (<1500 neutrophils/μl) found that the longitudinal incidence per 100 person-years of exposure was 3.8% and of severe neutropenia it was 0.9% (defined as <500/μl ≙ agranulocytosis according to AMSP criteria). The incidence of clozapine-induced neutropenia-related deaths was 0.013% (Myles et al. 2018). In our study, the incidence of agranulocytosis-related deaths amongst all patients treated with clozapine was 0.005% and the lethality of clozapine-induced agranulocytosis was 4.9%. No deaths were associated with neutropenia, so the lethality of both ADRs together was 3.0%. Agranulocytosis and neutropenia occurred significantly more frequently with single imputation of clozapine than in combination with other drugs. Other studies also did not attribute polypharmacy including clozapine to a higher ADR risk (van der Horst et al. 2023). Our results support the general consensus that agranulocytosis can occur at low doses (Choudhury et al. 2021), for example during the titration phase. Unlike many other ADRs, higher age does not appear to be a risk factor for clozapine-induced neutropenia and agranulocytosis in our study. This result is contrary to findings of other authors who found higher age to be associated with higher risk for neutropenia and agranulocytosis (Alvir et al. 1993; Balda et al. 2015; Lorenzo-Villalba et al. 2020). While we found that women were significantly more frequently affected by blood count changes than men, a more recent study from 2016 challenges this notion suggesting women had a lower risk of clozapine-associated dyscrasias (Demler et al. 2016). Due to these inconsistent findings, altered monitoring intervals according to age or sex are currently not available. 

#### Severe weight gain

The propensity of SGAs to cause weight gain is perhaps one of their most worrisome ADRs, especially because of the long-term health implications (Doane et al. 2022). While clozapine’s risk of weight gain is exceeded for example by olanzapine, it still remains an antipsychotic with one of the highest risks of this ADR (Schneider et al. 2020). The current study found a frequency of clozapine-induced severe weight gain of 0.089%, which is considerably lower than in comparative studies: In a retrospective study on weight gain under clozapine treatment the cumulative incidence of patients who became significantly overweight during clozapine treatment was over 50% (Umbricht et al. 1994). A study from 2010 found a mean increase in weight of 2.62 kg/week in 72.41% of Asian patients treated with clozapine (Mahendran et al. 2010). The much lower incidence of weight gain in our study may be the result of the inpatient setting of our study: While antipsychotic-induced weight gain begins early during treatment, it may continue over a longer period of time (i.e., after discharge). Moreover, patients treated with clozapine may already be overweight or have gained weight due to previous treatment with antipsychotics (Doane et al. 2022), perhaps making weight gain less apparent or more difficult to attribute to clozapine. This fact and, as will be discussed in more detail below, the underreporting of ADRs should also be considered.

#### Gastrointestinal disorders

Perhaps overshadowed by agranulocytosis, clozapine’s propensity to impair gastrointestinal motility is often overlooked (Shirazi et al. 2016; Cohen 2017). This effect is most likely due to clozapine’s anticholinergic, antihistaminergic, and anti-serotonergic properties resulting in the slowdown of intestinal peristalsis (Xu et al. 2021). An observational study including 188 patients found a significantly higher incidence of 2.12% of constipation and ileus during treatment with clozapine (Ingimarsson et al. 2018) compared to our study (0.05%). Three of the 5 documented deaths under clozapine treatment were attributable to this ADR, the (sub-)ileus therefore was the most lethal ADR of clozapine in our study, even more lethal than agranulocytosis. Cohen compared studies on the occurrence of clozapine-induced gastrointestinal hypomobility and agranulocytosis. He also found a higher mortality rate for gastrointestinal hypomobility (15–27.5%) than for agranulocytosis (2.2–4.2%) (Cohen 2017). This alarming finding calls for new standards in monitoring for the (sub-)ileus in patients treated with clozapine. Moreover, it is imperative to avoid the concomitant use of other drugs with strong anticholinergic properties in patients treated with clozapine whenever possible as this can further increase the risk of ileus: In both cases of fatal clozapine-induced ileus in our study, an array of other anticholinergic drugs such as pirenzepine were used. High clozapine dose is known to be a risk factor for the development (Palmer et al. 2008) and mortality (West et al. 2017) of clozapine-induced ileus. Although anticholinergic properties appear to have dose-dependent effects (Lavrador et al. 2021), the results of our study emphasize monitoring patients even with lower dose, as the median dose in patients with an ileus was “only” 262 mg/day and therefore lower than in other ADRs such as seizures.

#### Delirium

Delirium was the second most commonly observed clinical presentation of clozapine-induced ADRs in the present study. A study on 139 psychiatric inpatients from 2003 suggested that delirium occurs in up to 10% of inpatients treated with clozapine and particularly affects older patients (Centorrino et al. 2003). While our incidence of delirium was extensively lower, our study also confirms the aforementioned age-dependent effect. Moreover, we found that the risk of delirium was higher in patients treated with multiple drugs, confirming the frequent assumption that polypharmacy increases the risk of delirium (Kurisu et al. 2020). In our study, delirium was particularly often associated with risk factors (72.5% of all delirious patients). This correlation has also been reported by other authors (Pisani et al. 2002). One reason for the high number of risk factors could be the comparatively higher age of the patients with clozapine-associated delirium, pre-existing brain damage (such as dementia), and a more complex medical history including a longer medication list.

#### Extrapyramidal symptoms

Upon its discovery, clozapine was initially considered devoid of EPS (Beckmann et al. 1979). Even now, only a few descriptions and/or case reports of EPS under clozapine treatment are available (Cohen et al. 1991; Grover and Sahoo 2015). Although also rare in this study, EPS—almost exclusively in form of a Pisa syndrome—could be attributed to clozapine alone in a total of 8 cases and in 24 cases, clozapine was at least one of the drugs involved. In so, EPS under clozapine occurred primarily in combination with other drugs. When using therapeutic doses of clozapine, it occupies 40–50% of D2 receptors and is therefore generally too low to induce EPS (Gerlach et al. 1996) for which the threshold lies at 80% D2 receptor occupancy (Siafis et al. 2023). When combining clozapine with other (antipsychotic) drugs with antagonist properties at D2 receptors, additive D2 receptor antagonism may induce or aggravate EPS. However, the administration of clozapine can also lead to an improvement in antipsychotic-induced EPS (Wong et al. 2022). These heterogenous effects of clozapine in causing or improving EPS render the matter inconclusive for now and call for further research. We conclude that regular monitoring of EPS—even in patients treated with clozapine—should not be neglected.

#### Strengths and limitations

AMSP enables the detection of even rare ADRs due to its nature as a structured multicentral drug monitoring program with an observation period of 23 years of almost half a million psychiatric inpatients. Therefore, it reflects a particularly realistic patient setting. The detection of ADRs was made clinically rather than with inclusion of determined research criteria. This is certainly an advantage for the transferability of the results to everyday care, as a “real-life” patient collective was examined, rather than a highly selected one. Unlike naturalistic studies in outpatient settings that are often only able to assess prescription rates, AMSP is able to assess actual drug utilization rates. Moreover, all ADRs recorded by AMSP are vigorously discussed allowing us to determine causality of adverse events and drugs with high certainty.

However, the study has some limitations that must be considered while interpreting the results. The data only includes inpatients from hospitals in German-speaking countries. The results may therefore not be generalizable to outpatient settings or treatment practices on an international level. Drugs such as clozapine may have a different spectrum and/or frequency of ADRs at the beginning of treatment or when adjustment such as changes in dose or concomitantly used drugs are made than in long-term outpatient-treatment or after discharge. Psychiatric inpatients are generally more severely ill and may suffer from a higher degree of comorbidity, which can influence the type and frequency of ADRs. The analysis of the risk factors for the development of a clozapine-associated ADR is limited to the patients who developed an ADR because AMSP only records the risk factors for these patients but not for all patients, so we cannot make any comparisons here. The incidence of many ADRs appears to be significantly lower compared to other studies, a reason being the sole recording of severe ADRs and AMSP’s strict inclusion criteria. Additionally, many adverse effects occur with unspecific symptoms, which may have been incorrectly assessed as not drug related. Moreover, ADRs such as sedation and weight gain are documented much less frequently than ADRs that may seem more dangerous at first glance, such as agranulocytosis or epileptic seizures. The AMSP program is an induced spontaneous reporting system, which cannot have the same reliability in recording or reporting ADRs as a clinical trial can. Bias due to inadequate reporting must be considered as individual clinicians report ADRs in addition to their regular work according to their individual resources, motivation and evaluation habits. Depending on the time, motivation and financial resources of the participating hospital, individual and institutional bias in the sense of underreporting cannot be ruled out and is difficult to combat. To reduce this risk and to ensure that data is as objective as possible, all ADRs were thoroughly analyzed prior to inclusion in the AMSP database. It can therefore be assumed that the validity of the data is high.

## Conclusion

To our knowledge, the present study is the first to assess severe clozapine-associated ADRs in such a large sample of inpatients (*N* = 38,349 patients). The results of this study highlight the eminent importance of alertness and appropriate monitoring during treatment with clozapine. While much attention is paid to clozapine’s most prominent ADR, agranulocytosis, for which a mandatory monitoring has long been established, other perhaps even more dangerous ADR such as ileus should not be underestimated. Knowledge about the possible occurrence of ADRs under clozapine treatment, especially about the frequently occurring severe ones, is essential for everyday treatment. We recommend paying special attention to frequently overlooked symptoms such as constipation, as clozapine-associated ileus appears to have a particularly high mortality. In so, abdominal examinations, including exanimation of the intestinal peristalsis, should regularly be performed. Though they are easy to perform and low in cost, this type of examination is often neglected in clinical practice compared to laboratory tests, EEGs, and electrocardiograms. This can have fatal consequences for the patients’ health and can even lead to death. Further prospective real-world data in treatment-resistant schizophrenia is needed to assess both inpatient and outpatient drug safety quality when initiating and managing clozapine treatment. Overcoming barriers to clozapine underutilization is substantially established when maximizing drug safety in clozapine-treated patients.

### Supplementary Information

Below is the link to the electronic supplementary material.

Supplementary file1 (DOCX 20 KB)

## Data Availability

The datasets referred to and/or analyzed in the present study are available from the corresponding author on reasonable request.
