# Supplementary material for: Clozapine-associated adverse drug reactions in 38,349 psychiatric inpatients: drug surveillance data from the AMSP project between 1993 and 2016
Source: J Neural Transm (Vienna). 2024 Aug 13;131(9):1117–34. doi: 10.1007/s00702-024-02818-7 (PMC11365862; doi:10.1007/s00702-024-02818-7)
Supplement: Supplementary file 1 — Supplementary file1 (DOCX 20 KB) [file 702_2024_2818_MOESM1_ESM.docx]

***Table 1****: Comparison of different antipsychotic drugs regarding their associated ADRs*

|  | **Number of patients treated** | **All cases of AP-associated ADRs** | | | **ADRs with single imputation of APs** | | |
| --- | --- | --- | --- | --- | --- | --- | --- |
|  |  | **All ADR-cases** | **Frequency in relation to all patients treated with the respective AP in %** | **ADR rate of the respective drug compared to ADR rate of CLZ, p-value** | **ADR-cases with “single imputation” of the AP** | **Frequency in relation to all patients treated with the respective AP being “imputed alone” in %** | **ADR rate of the respective drug compared to ADR rate of CLZ, p-value** |
| **All AP** | 333,175 | 3591 | 1.08 |  | 1928 | 0.58 |  |
| **Low potency FGAs** | 102,469 | 469 | 0.46 |  | 79 | 0.08 |  |
| **Pipamperone** | 24,117 | 117 | 0.49 | <0.001 | 38 | 0.16 | <0.001 |
| **Melperone** | 18,984 | 56 | 0.29 | <0.001 | 9 | 0.05 | <0.001 |
| **Chlorprothixene** | 14,018 | 70 | 0.50 | <0.001 | 12 | 0.09 | <0.001 |
| **Prothipendyl** | 15,742 | 58 | 0.37 | <0.001 | 9 | 0.06 | <0.001 |
| **Promethazine** | 17,545 | 73 | 0.42 | <0.001 | 11 | 0.06 | <0.001 |
| **High potency FGAs** | 92,980 | 851 | 0.92 |  | 259 | 0.28 |  |
| **Haloperidol** | 37,650 | 395 | 1.05 | <0.001 | 165 | 0.44 | <0.001 |
| **Flupentixol** | 10,823 | 78 | 0.72 | <0.001 | 32 | 0.30 | <0.001 |
| **Perazine** | 15,496 | 148 | 0.96 | <0.001 | 62 | 0.40 | <0.001 |
| **SGAs including clozapine** | 226,161 | 2786 | 1.23 |  | 1590 | 0.70 |  |
| **Clozapine** | 38,349 | 586 | 1.53 |  | 396 | 1.03 |  |
| **SGAs without clozapine** | 187,812 | 2200 | 1.17 |  | 1194 | 0.64 |  |
| **Olanzapine** | 54,822 | 722 | 1.32 | 0.007 | 421 | 0.77 | <0.001 |
| **Quetiapine** | 66,209 | 500 | 0.76 | <0.001 | 223 | 0.34 | <0.001 |
| **Risperidone** | 51,683 | 582 | 1.13 | <0.001 | 314 | 0.61 | <0.001 |
| **Amisulpride** | 14,168 | 223 | 1.57 | 0.705 | 138 | 0.97 | 0.552 |
| **Aripiprazole** | 15,988 | 148 | 0.93 | <0.001 | 98 | 0.61 | <0.001 |

^N = number (of cases), ADR = adverse drug reaction; AP(s) = antipsychotic(s), FGAs = first-generation antipsychotics, SGAs = second-generation antipsychotics, CLZ = clozapine^
